# Analysis of photosynthetic and water-use efficiency of *piper aduncun *in a degraded area from gaseous exchange

**DOI:** 10.1186/1753-6561-8-S4-P129

**Published:** 2014-10-01

**Authors:** Rebeca P Guimarães, Vinícius P Duarte, Breno R Santos, Thiago C de Souza

**Affiliations:** 1 Universidade Federal de Alfenas, Alfenas, Brazil

## Background

Plants are able to survive in a distinctive environment from its usual one, especially due to morphological or physiological changes, thus a good ability of acclimatization and phenotypic plasticity is present [1]. Light and water availability are decisive factors in a plant's survival, the occurrence of those is irregular, because luminosity is broadly available on the edges and large clearingsaccording to the criteria proposed by Souza, et al.[3] and scarce inside woods [2] and water availability depends on local pluviosity. Considering this, studies relating photosynthesis and water-use efficiency to phenotypic plasticity are major to the discovery of species capable of tolerating degraded environments with diverse luminous and pluvious intensity, thus, contributing to the enhancement of them. Based on the given information, this study aims to look into the behavior of these variables in *Piper aduncum *through gas exchange in distinctive spots of a gully.

## Methods

The study was performed at a rural property in Alfenas-MG between the months of January-April 2013. The local vegetation found on the region is semi-deciduous with little native species.

The gas exchange parameter was measured through a portable photosynthesis gas exchange system (IRGA, Model LI-6400XT, Li-Cor, Lincoln, Nebraska, USA). All measures were made through the morning between 8AM and 11AM, on a fully expanded leaf area and with due phytosanitary measures.

The evaluated parameters were: foliar photosynthetic rate (A) and the water-use efficiency (A/gs). The measures were taken on a foliar area of 6cm², the chamber's air flow had a CO_2 _concentration of 380 µmol mol^-1 ^. The air was collected through outside of the gully and then transported inside a protected container and then pumped to the chamber. A *photosynthetic photon flux *d*ensity (PFFD) of *800 µmol.m^-2^.s^-^1was used from an artificial light source (LI-6400-02B RedBlue LED, Li-Cor). The temperature was kept at 28ºC. The experimental designs were entirely randomized with 3 treatments (area of the edge, slope and the gully's bottom) with twelve repetitions. Averages and standart errors were done for data analysis.

## Results and conclusions

In this context, the study has shown that the photosynthetic rate was bigger on the edges of the gully (11,37 µmol.m^-2^.s^-1 ^ ± 1,06) and the water-use efficiency on the edges and the slope were equal. The difference between the treatments results from the slope and the gully's bottom can be explained, due to the fact that when the data was collected, sunlight rays passed through the canopy and despite that usually happening in a short amount of time, it can contribute actively to the photosynthetic photon flux, and thus, increasing photosynthetic activity [4]. The gully bottom showed lower water-use efficiency, therefore photosynthesis was small in relation to stomatal conductance.

Results lead to conclude that *Piper aduncum*demonstrates behavior prone to acclimatization and consequently of phenotypic plasticity when exposed to diverse luminousand water intensity from different spots of a degraded area.
